# Energy–structure–property relationships in uranium metal–organic frameworks

**DOI:** 10.1039/d3sc00788j

**Published:** 2023-04-06

**Authors:** Sylvia L. Hanna, Omar K. Farha

**Affiliations:** a Department of Chemistry and International Institute for Nanotechnology, Northwestern University Evanston IL 60208 USA o-farha@northwestern.edu; b Department of Chemical and Biological Engineering, Northwestern University Evanston IL 60208 USA

## Abstract

Located at the foot of the periodic table, uranium is a relatively underexplored element possessing rich chemistry. In addition to its high relevance to nuclear power, uranium shows promise for small molecule activation and photocatalysis, among many other powerful functions. Researchers have used metal–organic frameworks (MOFs) to harness uranium's properties, and in their quest to do so, have discovered remarkable structures and unique properties unobserved in traditional transition metal MOFs. More recently, (*e.g.* the last 8–10 years), theoretical calculations of framework energetics have supplemented structure–property studies in uranium MOFs (U-MOFs). In this Perspective, we summarize how these budding energy–structure–property relationships in U-MOFs enable a deeper understanding of chemical phenomena, enlarge chemical space, and elevate the field to targeted, rather than exploratory, discovery. Importantly, this Perspective encourages interdisciplinary connections between experimentalists and theorists by demonstrating how these collaborations have elevated the entire U-MOF field.

## Abstract 2

Energy–structure–property relationships in uranium metal–organic frameworks offer a deeper understanding of their behaviors, explaining why structure produces function. This understanding becomes crucial for the rational design of such materials with enhanced properties.

## Graphical abstract

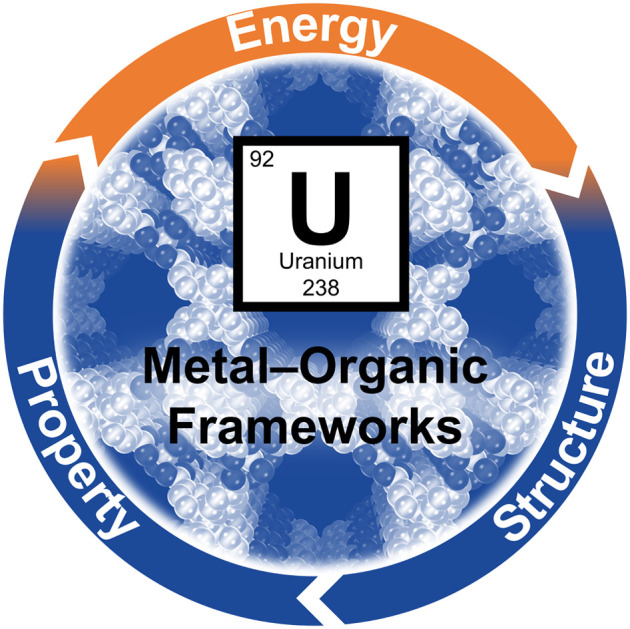

## Introduction

Among the diverse chemistry that the periodic table offers, uranium stands distinctly apart from s-, p-, and d-block metals, and even from its actinide and lanthanide relatives. With three f-electrons and unique electronic structure,^1^ uranium's character is complex and multifaceted. Indirect relativistic orbital expansion endows uranium with diverse oxidation states ranging from U(i) to U(vi),^2–6^ resulting in impressive redox activity.^7–9^ High coordination numbers and multiple bonding characterize uranium's coordination, and relativistic effects also change the extent of its bonding covalency.^7,10,11^

Consequently, these fundamental singularities amplify into the striking properties of uranium's molecular species and materials. Perhaps its most notable function, uranium shows promise for challenging reactions relevant in the highly industrialized Haber–Bosch and Fischer–Tropsch processes.^12–14^ Uranium's ability to activate small molecules is not only limited to N_2_ and CO, but also extends to other environmentally relevant species including CO_2_, NO, and hydrocarbons.^7^ Additionally, uranium possesses powerful photoredox abilities,^15–17^ impressive single molecule magnetism,^17–19^ and burgeoning catalytic capacity.^20–22^

In addition to its chemical properties, uranium's radioactive and energetic fission properties were unearthed over a century after the element's discovery by Martin Klaproth.^11^ Use of fissile uranium radioisotopes during the Manhattan Project cast a historically negative image on uranium, with deep scientific and societal implications. This image, paired with actual or perceived fear regarding its safety, is responsible in part for the relatively underdeveloped nature of scientific research on uranium, compared to transition metals. Currently, pressing demands within nuclear stockpile stewardship^23^ and the nuclear energy sector^24^ call for further development of uranium, made possible primarily through the scientific study of the ^238^U isotope.^25^

We broadly focus this Perspective on the further development of uranium through the study of uranium's crystal chemistry. Specifically, researchers have harnessed unique attributes of uranium by installing it in nanoscale hybrid materials called metal–organic frameworks (MOFs) which possess directional, mathematically predictable bonding patterns.^26–29^ Built from the self-assembly of organic, multitopic linkers and uranium-based single-atom or cluster nodes, uranium MOFs (U-MOFs) are a twist on their classical transition metal-based counterparts. Like traditional transition-metal MOFs, U-MOFs boast crystallinity, higher-order dimensionality, synthetic tunability, and impressive porosity. However, electronic, architectural, and behavioral differences make U-MOFs fundamentally distinctive.^30–32^ Importantly, U-MOFs provide valuable insight into the intersection of environmental stewardship and nuclear fuel processes, as their chelation environments mirror that of the uranium mineral, studtite.^33^ Additionally, U-MOFs possess optimal attributes to harness and develop uranium chemistry in a relatively facile manner; spatially separated nodes discourage ever-present disproportionation, and facile hydrothermal/solvothermal MOF syntheses allow researchers to tune the ligand environment without re-developing complex organometallic syntheses. Furthermore, U-MOFs can function as tailored waste forms for radioactive waste streams, allowing researchers to simultaneously recycle uranium and remove toxic chemicals.^34^ We refer to this broad class of materials as uranium MOFs (U-MOFs) for the remainder of this Perspective, but we note that they are also referred to as uranium–organic frameworks/compounds^34–41^ or uranyl/uranium coordination polymers^42–47^ in the literature.

**Scheme 1:**
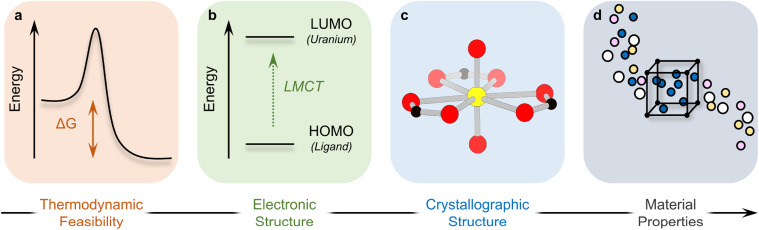
Structure of this Perspective, progressing from fundamental energetic characterization of U-MOFs to structures and resulting properties.

Most commonly, U-MOFs crystallize as hexavalent uranium polyhedra^48,49^ connected by organic linkers. In its hexavalent state, uranium typically exists as the linear, symmetric uranyl [UO_2_]^2+^ dication (Scheme 1c) where U(vi) binds to two axial oxygen atoms with short 1.8 Å bonds. The equatorial plane remains available for binding to carboxylate,^31^ phosphonate,^50^ imidazolate, and other groups, forming bipyramidal polyhedra.^32,42,43,51–54^ The hydrolysis of uranium can produce nodes with a plethora of nuclearity, and less-common node motifs comprised of clusters or lower-valent uranium also occur. Advancement of U-MOF underlying design principles has led to a library of impressive hybrid structures and unprecedented arrangements.^32,35,55,56^ This structural collection boasts emerging properties applied in catalysis, photochemistry, waste capture, electronics, sensing, non-linear optics, and luminescence. Importantly, ties between structure and property have been crucial to the progress of the U-MOF field from exploratory synthesis to more targeted development.^30,36,37,42,57–63^

A third dimension of exploration remains – that of energy. While structure–property relationships correlate the physical arrangement of atoms in a U-MOF lattice to the resulting material behavior, fundamental energetic investigations (thermodynamics, thermochemistry, theoretical calculations investigating structural and electronic configurations, *etc.*) allow researchers to *rationalize* these correlations. Energy–structure–property relationships thereby offer a deeper understanding of phenomena, explaining *why* structure produces function. This understanding becomes crucial for the rational design of targeted U-MOF materials with specific and enhanced properties that are energetically accessible. Indeed, energy–structure–property relationships ultimately offer a very precise knob to not only fine-tune existing material properties through structure, but to also discover next-generation materials with novel properties.

Over the last 8–10 years, the U-MOF field has begun to investigate energy–structure–property relationships. This Perspective serves to highlight the impact and importance of newly established energy–structure–property relationships in the development of U-MOFs. We structure this Perspective to progress from the energetic characterization of U-MOFs to the ways in which energy affects structure, and finally to the resulting connections between energy, structure, and property. We begin by examining the thermodynamics and thermochemistry of the energy landscape on which U-MOFs lie (Part I, Scheme 1a): are the targeted structures energetically accessible? Next, we discuss the electronic structure and properties of energetically feasible U-MOFs (Part II, Scheme 1b). Part III moves from theoretical calculations on U-MOF electronic structure to crystallographic structure and explains how structural distortions and geometries originate in energetic phenomena (Scheme 1c). In Part IV, we consider how energetics inform properties (Scheme 1d). Finally, we discuss the future potential of energy–structure–function maps as a method to harness knowledge as the field ages. As an underlying theme, this Perspective emphasizes the interdisciplinary relationship between experiment and calculation and is not designed to alienate either audience, but rather to enhance the connection between the two.

## Part I. Energetic accessibility of U-MOFs

Emergent U-MOF structures and properties depend upon the energetic feasibility of constructing the structure to begin with (Scheme 1a). While both thermodynamic and kinetic drivers impact framework formation, the U-MOF field has focused primarily on investigating the former. Calculated free energies of formation thus provide valuable insight into the thermodynamic stability of desired U-MOF products relative to their starting forms. This information in turn assists in predicting and explaining our power to synthetically access specific ligand and node motifs or even entire topologies.

**Fig. 1:**
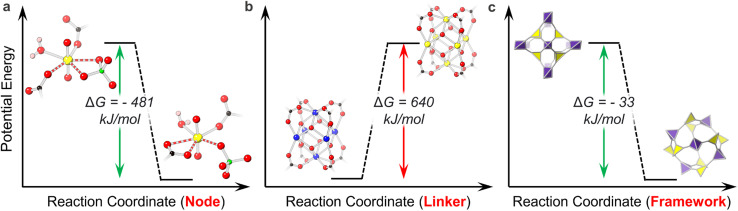
Thermodynamic favorability of (a) ligand motifs in ref. 53: URCP3 on the left and URCP4 on the right. Bonds of interest are dashed; (b) node motifs in ref. 55: Zr_6_ cluster on the left and U_6_ cluster on the right; (c) framework topologies in U-MOFs in ref. 57: NU-1306 on the left and NU-1305 on the right. O is shown in red, U in yellow, C in black, H in pink, S in green, and Zr in blue.

For example, Li *et al.* reported the single-crystal-to-single-crystal transformation of the U-MOF URCP3 to URCP4 and calculated that ligand coordination in the URCP4 isomer favored its relative stability. Both isomers crystallize with a uranyl node, a pseudorotaxane cucurbit[6]uril-based linker, and a sulfate anion (derived from the uranyl sulfate starting material). However, while URCP3 possesses a monodentate linker and bidentate sulfate, URCP4 holds a bidentate linker and monodentate sulfate. The thermodynamic stability of the URCP4 ligand binding motif over that of its isomer explains the irreversible and spontaneous transformation of URCP3 to URCP4 (Fig. 1a).^44^ Thermodynamics of ligand binding can also be observed in the study of Ejegbavwo *et al.* to post-synthetically install capping linkers into U_6_-Me_2_BPDC-8 (Me_2_BPDC^2−^ = 2,2′-dimethylbiphenyl-4,4′′-dicarboxylate). Theoretical calculations supported observed linker installation in the isostructural Th-based MOF and predicted the energetic feasibility of U_6_-Me_2_BPDC-8 transformation to a 10-connected (−308 kJ mol^−1^) or 12-connected (−641 kJ mol^−1^) MOF.^64^

In addition to ligand crystallization, the composition and structure of uranium nodes also depend on their energetic accessibility. The Shustova group demonstrated this concept during their efforts to extend MOF modularity by integrating actinide ions through transmetalation. Interestingly, attempted transmetalation of a Zr_6_ cluster UiO-type MOF (Zr_6_-Me_2_BPDC-8) with actinides proved unsuccessful (attributed to the lack of flexibility in the rigid MOF), while transmetalation of the analogous U_6_-Me_2_BPDC-8 MOF with Th resulted in Th_5.65_U_0.35_-Me_2_BPDC-8 and marked the first actinide-to-actinide cation exchange in MOFs. Density functional theory (DFT) calculations revealed an unfavorable energy to substitute six Zr node atoms to U (Fig. 1b); thus favorable substitution energy for U to Th likely allows for its successful transmetalation.^65^ The use of U as a surrogate for Np motivated Saha and Becker to similarly investigate the energetic favorability of Np incorporation into U-MOFs. Computational studies suggested thermodynamically favorable incorporation of Np, even in the face of changing node geometry.^66^

Combining the effects of U-MOF linker and node motifs with framework topology reflects overall lattice stability. In collaboration with the Hendon group, our group used total energy calculations paired with experimental studies to quantify the synthetic feasibility of a metastable U-MOF isomer, NU-1306. Isomers NU-1305 and NU-1306, both comprised of a tetrakis(4-carboxyphenyl)methane linker and mononuclear uranyl node, crystallized in **ctn** and **bor** topologies, respectively. We identified the thermodynamic stability of NU-1305 over NU-1306 (Fig. 1c) and demonstrated how these energetic phenomena allowed conversion from metastable NU-1306 to globally stable NU-1305, and *vice versa*.^67^

## Part II. Effect of U-MOF electronic structure on coordination and bonding

Once envisioned U-MOF structures become synthetically feasible, researchers resolve their electronic properties by characterizing the energy levels of the frontier molecular orbitals involved in electronic transitions. Simulated DFT calculations compliment absorption, infrared, Raman, fluorescence, and photoluminescence spectroscopy to elucidate the fundamental nature of bonding in U-MOFs. Ultimately, these studies demonstrate how f- and/or d-electrons affect U-MOF material properties.

Frontier molecular orbitals in uranyl-based U-MOFs most often possess a highest occupied molecular orbital (HOMO) with primarily ligand character. Specifically, phenyl- or benzene-based π character^68,69^ and 2p orbitals from carboxylate oxygen atoms^70–73^ dominate this energetic regime. The lowest unoccupied molecular orbital (LUMO) is primarily stabilized by uranium and exhibits 5f character^68,70–75^ or uranyl d-orbital qualities.^69,76^ Ligand to metal charge transfer (LMCT) in these compounds point to electronic promotion from organic linker to uranium center and is often indicated by low energy absorption bands (Scheme 1b).^76,77^ Calculated natural charges in electronically active frameworks also reflect LMCT behavior: while the natural charge of the free uranyl cation is expected to be 2.81, bound uranyl cations in MOFs show values in the range of 1.38–1.54.^46,70,78,79^ Pandey *et al.* published a detailed study systematically exploring DFT methods to distinguish the contribution from organic and inorganic components to the HOMO–LUMO band gap origin in U-MOFs.^80^

Since relativistic effects strongly influence uranium bond covalency, U-MOF electronic structures closely relate to the ionic *vs.* covalent nature of linker-to-node bonding. In general, organic linkers predominantly exhibit covalent bonding characteristics while the metallic uranyl node demonstrates ionic bonding characteristics.^71^ Bond order and electron density calculations of axial (U-oxo) and equatorial bonds in the uranyl subunit closely link to their specific character. Calculated and experimental bond lengths for uranyl-oxo bonds in U-MOFs exhibit double bond (2.0–2.1)^46,47,72,81^ or partial triple bond (2.20–2.37)^73,77,82^ character. These axial bonds are classified as covalent bonds, justifying their relatively inert behavior compared to U–O equatorial bonds. U-oxo bonding character in MOFs can, however, be influenced by the presence of other interacting ions.^79,83–85^ Equatorial U–O bonds predominantly exhibit smaller bond orders (0.3–0.6) which suggest mostly ionic or weak covalent character.^46,47,72,77,81,82,86^

These electronic structure considerations directly affect U-MOF bonding features and coordination behavior. For example, in a variety of uranyl-based MOF systems containing both U–O and U–N equatorial bonds, stronger interactions with uranium originate from oxygen-bound rather than nitrogen-bound ligands.^47,69,81^ While this behavior corresponds with uranium's well-known oxophilicity, it also reflects the stronger LMCT of carboxylic acid-based linkers over nitrogen-bound linkers like phenanthroline.^46^ The Sun group also demonstrated effects of the HOMO–LUMO gap in their uranyl-based MOF system.^78^ While pairing the uranyl node with a terpyridine-based metalloligand produced infinite 1D chain structures, addition of the auxiliary 4,4′-biphenyldicarboxylic acid linker resulted in 3D catenated frameworks. Interestingly, increasing dimensionality from 1D to 3D systems decreased the HOMO–LUMO gap from 4.29 eV to 2.93 eV, suggesting that addition of the auxiliary ligand resulted in more diffuse electronic motion.

## Part III. Energetic origins of crystallographic U-MOF configurations

The influence of energetics on U-MOF crystallization impacts resulting material properties dramatically. Because structural distortions, unusual geometries, and unique bonding originate in energetic phenomena or instability, theoretical calculations provide insight into the nature of these crystallographically characterized configurations. Within uranyl-based MOFs (Scheme 1c), energetic effects influence axial U-oxo bonds, equatorial node bonds, and overall crystallization preference.

**Fig. 2:**
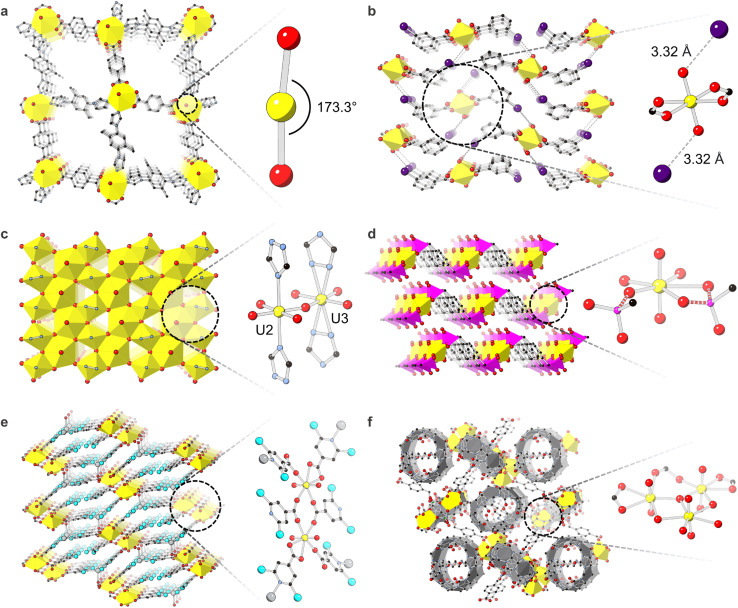
Crystal structures demonstrating (a) distortion of axial U–O bonds in ref. 70. Only one of the structure's three interpenetrated lattices is shown for clarity; (b) axial U–O bond interaction with iodine from the *m*-iodobenzoic acid linker in ref. 76; (c) Kagome lattice and equatorial U–O bonding modes in ref. 81; (d) equatorial bonding of phosphonate-based linkers to uranyl in [NH_4_]_2_[UO_2_(pmb)] from ref. 82. NH_4_ is removed from the structure for clarity, and phosphonate linkers are terminated at carbon atoms. Bonds of interest are dashed; (e) U-Ag-2,6-DCPCA containing Ag–N bonds from ref. 62; (f) compound 1 from ref. 79 where *N*,*N*′-bis(4-cyanobenzyl)-1,4-diammoniobutane dinitrate are bound to an asymmetric trinuclear uranyl node and are encapsulated by cucurbit[6]uril shown in grey panels. O is shown in red, U in yellow, C in black, S in green, I in navy, N in light blue, P in pink, Cl in aqua, and Ag in grey. H are hidden for clarity.

For example, Chen *et al.* reported a U-MOF containing unusual 173.3° curvature in the typically linear 180° uranyl dication (Fig. 2a).^79^ The 8-coordinate uranyl unit was bound to two bidentate 1,4-benzenedicarboxylic acid linkers and two monodentate 1-(4-(1*H*-imidazol-1-yl)-2,5-dimethylphenyl)-1*H*-imidazole linkers. DFT investigations revealed that the electronegative heterocyclic imidazole units induced the bent uranyl geometry by generating higher charge populations in the valence U 6d shell. Studies by the Cahill group on harnessing terminal oxo chemistry revealed energetic foundations of both non-covalent and covalent oxo interactions in U-MOFs. In one case, 1D chains of the uranyl cation bound to benzoic acid, *m*-chlorobenzoic acid, *m*-bromobenzoic acid, or *m*-iodobenzoic acid non-covalently assembled through hydrogen or halogen-oxo interactions (Fig. 2b). Through DFT calculations, the relative strength of these non-covalent halogen-oxo interactions was shown to originate in inductive effects and halogen polarizability.^84^ Conversely, covalent oxo-Ag^+^ interactions in a separate U-MOF decreased uranyl bond orders through electron donation from Ag^+^ to U-oxo σ- or π-antibonding orbitals.^85^

Equatorial U–O bonding modes and distortions also originate in energetic roots.^87^ In their report of the first f-element Kagomé topology coordination complex U^V^O(U^VI^O_2_)_2_(OH)_5_(Triaz)_2_ (where Triaz = 1,2,4-triazolate), Smetana *et al.* observed disorder between two complementary mutually excluding U positions. Bound monodentately by triazolate-based linkers, U2 occupied 84.6%, and U3, bound in a bidentate fashion, occupied 15.4% (Fig. 2c). The nature of this disorder was clarified through DFT studies on triazolate linker interactions; strong repulsions between triazole nitrogen atoms and nearby oxygen atoms prevented bidentate triazole binding in U2 while additional hydrogen-bond stabilization in U3 compensated for N–O repulsions and encouraged bidentate triazole coordination.^88^ Theoretical calculations also helped distinguish binding motifs of equatorial groups in uranyl phosphonate compounds. For example, the 1,4-phenylenebis(methylene))bis(phosphonic acid) linker can bind to uranyl through both P–O^−^ and P=O motifs, driven by electrostatic forces or electron lone pair donation, respectively. The Wang group confirmed their assignment of these crystallographic binding modes in [NH_4_]_2_[UO_2_(pmb)] where pmbH_4_ = 1,4-phenylenebis(methylene))bis(phosphonic acid) through calculations of bond indices and electron density (Fig. 2d).^89^

Apart from specific geometries of axial U-oxo or equatorial U–O bonds, effects of energetics have also been observed on U-MOF overall structure. For instance, Mei *et al.* reported an energy decomposition analysis which identified an Ag–N bond as the driving force for the formation of the U-MOF, U-Ag-2,6-DCPCA (H-2,6-DCPDA = 2,6-dichloroisonicotinic acid) (Fig. 2e).^72^ Findings by the same group also directly related the unique weaving configuration of their polyrotaxane polythreaded U-MOF to the asymmetric coordination of its trinuclear node through quantum chemical calculations (Fig. 2f).^90^

## Part IV. Energetic origins of U-MOF structure–property relationships

A strong driver in the exploration of U-MOFs is the potential for discovery of novel properties (Scheme 1d). Indeed, the multifaceted character of uranium promises behaviors that transition metal-, lanthanide-, and even other actinide-based MOFs cannot provide.^7^ Chemical structures can explain or correlate to emergent properties, but the underlying reasons for their existence lie in energetic processes. The combination of theoretical calculations with experiment thereby describes why certain U-MOF structures produce properties such as spontaneous de-interpenetration, photochromism, radiation resistance, radioactive waste capture, and catalysis.^38,40,91^ This fundamental understanding of U-MOF behaviors not only explains chemical phenomena but also enhances material properties.

**Fig. 3:**
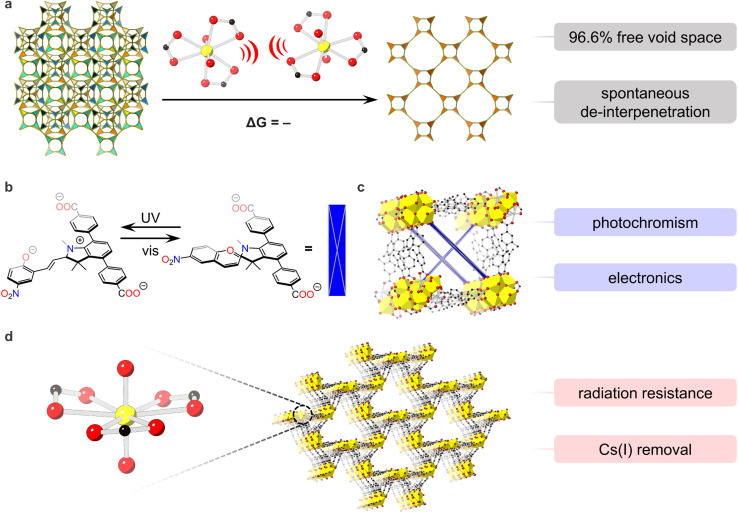
Energy–structure–property relationships in (a) the spontaneous de-interpenetration of NU-1303-6 in ref. 86. Charged point–point repulsions between nearby nodes on different interpenetrated lattices repel each other, causing spontaneous de-interpenetration; (b) photochromic spiropyran linker in (c) actinide-based framework in ref. 89; (d) U-MOF with umbellate distortions in ref. 90. O is shown in red, C in black, H are hidden for clarity. U is shown in yellow in all panels except for (c) where heterometallic nodes are comprised of Th_4.77_U_1.23._

One recent example of novel behaviors in U-MOFs is our group's discovery of spontaneous de-interpenetration – a property unobserved in any network material to date.^92^ De-interpenetration transformed NU-1303-6, a 6-fold interpenetrated U-MOF with 14.2 Å and 19.8 Å pores, into an open, single-lattice structure with 40.7 Å pores and record-high free void space (96.6%). This generation of porosity in the absence of external stimuli proves valuable for various applications including gas storage, catalysis, and electronics. Energetic investigations into the origins of this phenomena revealed that charged point–point repulsions between anionic uranyl nodes, present across the entire energy landscape, drove structural changes and reversed typical thermodynamic framework favorability (Fig. 3a).

U-MOFs also exhibit impressive optoelectronic properties,^39,93^ as seen in the first photochromic actinide-based framework, reported by the Shustova group.^94^ Using a photoswitchable spiropyran-based linker (Fig. 3b) paired with a heterometallic Th_5_U cluster node, Martin *et al.* accessed dynamically controlled conductivity and electronic properties. Importantly, electronic structure calculations revealed fundamental differences between photochromic Th_5_U MOFs and non-photoresponsive Th_5_U MOFs; frontier orbitals of the former involved in electronic transitions originated from U and Th 5f orbitals while those of the latter localized on the linker. In this way, photophysical properties in actinide MOFs were tied to electronic structure for first time linker (Fig. 3c).

U-MOFs also show value as adsorbent materials for fission product waste and contamination remediation. For example, the Wang group reported a U-MOF with impressive radiation resistance up to 200 kGy of γ and β irradiation and excellent chemical stability.^34^ Additionally, this U-MOF exhibited selective Cs(i) removal from aqueous solution with a distribution coefficient at the same order of commercial materials. These properties were attributed to the rare U-MOF structure, where 2D graphene-like sheets of [(CH_3_)_2_NH_2_][UO_2_(L2)]·0.5DMF·15H_2_O (L2 = 3,5-di(4′-carboxylphenyl) benzoic acid) catenate into a 3D framework with geometric distortions in the equatorial uranyl plane. Linear transit calculations further revealed that structural umbellate distortions were rooted in electronic behavior; repulsions from the umbrella-shaped equatorial carboxylate ligands pushed O_4_'s valence orbitals up in energy, producing a better orbital energy match with uranium's contracted 5f valence orbitals (Fig. 3d).

## Conclusions and outlook

Energy–structure–property relationships in U-MOFs enable a deeper understanding of chemical phenomena, enlarge chemical space, and elevate the U-MOF field to targeted, rather than exploratory, discovery. In this Perspective, we have detailed the impacts of theoretical calculations highlighting energetic phenomena on U-MOF thermodynamics, thermochemistry, electronic configuration, crystallographic structure, and material properties. We believe that energy–structure–property relationships lie at the heart of innovation and progress, and their detailed development will inspire the next generation of U-MOF materials with advanced properties. Importantly, close connections between experiment and calculation are crucial for this type of progress, particularly in the field of U-MOFs where much of uranium's promise remains undiscovered.

As the U-MOF field ages and the library of energetically characterized materials increase, concrete and organized connections between a material's structure, property, and energetic favorability become vital. Looking forward, we propose the eventual application of energy–structure–function maps in the U-MOF field.^95–100^ These maps rely on machine learning to reveal the energetically accessible regions of the system's lattice-energy surface and propose possible structures and properties for the building blocks of choice. Such a tool promises special value for exploring the multifaceted, complex, and unique crystal chemistry of uranium.

## Author contributions

S. L. H. conceived the manuscript idea, led the investigation, and wrote the manuscript. O. K. F. supervised the writing of this manuscript.

## Conflicts of interest

O. K. F. has a financial interest in NuMat Technologies, a startup company that is seeking to commercialize MOFs. All other authors declare no competing interests.

## Supplementary Material
